# Schwann Cell Precursors; Multipotent Glial Cells in Embryonic Nerves

**DOI:** 10.3389/fnmol.2019.00069

**Published:** 2019-03-26

**Authors:** Kristjan R. Jessen, Rhona Mirsky

**Affiliations:** Department of Cell and Developmental Biology, University College London, London, United Kingdom

**Keywords:** Schwann cell precursor, neural crest, nerve development, PNS, Schwann cell lineage, PNS glia, multipotent glia

## Abstract

The cells of the neural crest, often referred to as neural crest stem cells, give rise to a number of sub-lineages, one of which is Schwann cells, the glial cells of peripheral nerves. Crest cells transform to adult Schwann cells through the generation of two well defined intermediate stages, the Schwann cell precursors (SCP) in early embryonic nerves, and immature Schwann cells (iSch) in late embryonic and perinatal nerves. SCP are formed when neural crest cells enter nascent nerves and form intimate relationships with axons, a diagnostic feature of glial cells. This involves large-scale changes in gene expression, including the activation of established glial cell markers. Like early glia in the CNS, radial glia, SCP retain developmental multipotency and contribute to other crest-derived lineages during embryonic development. SCP, as well as closely related cells termed boundary cap cells, and later stages of the Schwann cell lineage have all been implicated as the tumor initiating cell in NF1 associated neurofibromas. iSch are formed from SCP in a process that involves the appearance of additional differentiation markers, autocrine survival circuits, cellular elongation, a formation of endoneurial connective tissue and basal lamina. Finally, in peri- and post-natal nerves, iSch are reversibly induced by axon-associated signals to form the myelin and non-myelin Schwann cells of adult nerves. This review article discusses early Schwann cell development in detail and describes a large number of molecular signaling systems that control glial development in embryonic nerves.

## Introduction

The cells of the neural crest often referred to as neural crest stem cells, give rise to a number of sub-lineages, one of which is the glial cells of the peripheral nervous system (PNS). This group of cells includes the myelin and Remak (non-myelin) Schwann cells in peripheral nerves, terminal Schwann cells at the neuromuscular junction, satellite glial cells in ganglia and enteric glial cells in the gut. This review article will focus on the appearance of the Schwann cell sublineage, the major PNS glial population and the one that has been most intensively studied.

**Figure 1:**
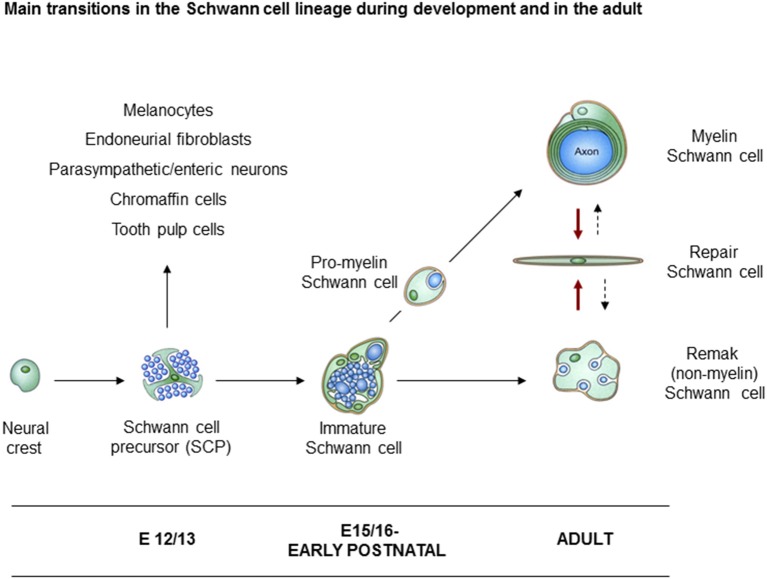
Main transitions in the Schwann cell lineage. A scheme illustrating key cell types involved in Schwann cell development, and in nerve repair. Also shown are the alternative cells that can develop from Schwann cell precursors (SCP) in addition to immature Schwann cells (iSch). Black uninterrupted arrows: normal developmental transitions. Red arrows: the Schwann cell injury response. Stippled arrows: post-repair formation of myelin and Remak cells (with permission from Jessen et al., 2015b).

In rodents, neural crest cells transform to Schwann cells of adult nerves through the generation of two well defined intermediate stages. These are the Schwann cell precursors (SCP) in early embryonic nerves, and immature Schwann cells (iSch) in late embryonic and perinatal nerves (Figure 1). The lineage therefore includes three main developmental transitions: (1) the formation of SCP from crest cells; (2) the generation of iSch from SCP; and (3) the mostly postnatal divergence of iSch to adopt the strikingly different phenotypes of myelin and Remak (non-myelin) Schwann cells (Jessen and Mirsky, 2005a; Jessen et al., 2015b; Monk et al., 2015). A fourth major phenotypic transformation potentially takes place in the adult. This is triggered by nerve injury which transforms myelin and Remak cells to repair Schwann cells, cells that are specialized to support nerve regeneration (Jessen and Arthur-Farraj, 2019; Jessen and Mirsky, 2016).

This review article will focus on the embryonic phase of Schwann cell development. We will discuss the phenotype and biology of SCP, the generation and the distinctive properties of iSch, and the signals that control survival, proliferation and lineage progression in this system.

## Outline of the Neural Crest Schwann Cell Lineage

### Schwann Cell Precursors (SCP)

SCP are found in mouse embryonic nerves at embryo day (E) 12–13 (rat E14–15). These cells have the characteristic phenotype of glial cells as we will discuss in the following section. Interestingly, SCP appears to retain the stem-cell-like feature of developmental multipotency, a cell biological feature that they share with early glial cells in the CNS, the radial glia (Pinto and Götz, 2007; Kriegstein and Alvarez-Buylla, 2009). SCP are closely integrated with axons in newly formed nerves and rely acutely on axonal signals for survival. Axons and associated SCP form the compact structures of embryonic nerves, from which connective tissue is excluded. Present evidence indicates that SCP are restricted to embryonic nerves, and these cells have not been shown to take part in the maintenance of adult nerves or in the response of peripheral nerves to injury.

### Immature Schwann Cells (iSch)

SCP form iSch that are present in mouse nerves from E15–16 (E17–18 in rat) extending to the perinatal period. iSch associate with connective tissue and basal lamina inside nerves, which at this stage also contain blood vessels, and do not depend on axons for survival. Starting around birth, these cells take part in a process termed radial sorting (Feltri et al., 2016). This generates Schwann cells in a 1:1 ratio with the larger diameter axons. In rodents, these cells are referred to as pro-myelin cells and they mostly go on to form myelin sheaths. In humans, however, many axons in a 1:1 ratio remain unmyelinated. The smaller axons do not take part in radial sorting, but gradually associate with iSch by taking up a position in shallow troughs along their surface, thus forming Remak bundles (Jessen and Mirsky, 2005a; Monk et al., 2015).

### Axonal Signals

Schwann cell development is to a striking degree dependent on and driven by, axon-associated signals. They ensure the survival of SCP and promote their differentiation, determine whether iSch differentiate as myelin or Remak cells and govern the highly specialized architecture and molecular expression of myelin Schwann cells. Signals from axons also drive the proliferation of developing Schwann cells until they fall out of division at the pro-myelin stage (Webster and Favilla, 1984). In undisturbed adult nerves, myelin and Remak cells are mitotically quiescent but transiently re-enter the cell cycle after nerve injury and in some pathological conditions (Chen et al., 2007; Stierli et al., 2018).

### Boundary Cap Cells

The present article deals with Schwann cell development as elucidated in studies on spinal nerves, such as the developing sciatic nerve. Other studies have focussed on Schwann cells, and other cellular derivatives that emerge from a distinct sub-population of crest cells, called boundary cap sells (Maro et al., 2004; Coulpier et al., 2009; Radomska and Topilko, 2017). Boundary cap derived Schwann cells are particularly important in ensheathing the axons of the spinal nerve roots. They have a distinct molecular signature, including early expression of the pan Schwann cell marker S100 and the transcription factor Krox20 (Egr2), which is a key regulator of myelination in peri- and post-natal nerves.

## Schwann Cell Precursors Show an Obvious Glial-Like Phenotype

**Figure 2:**
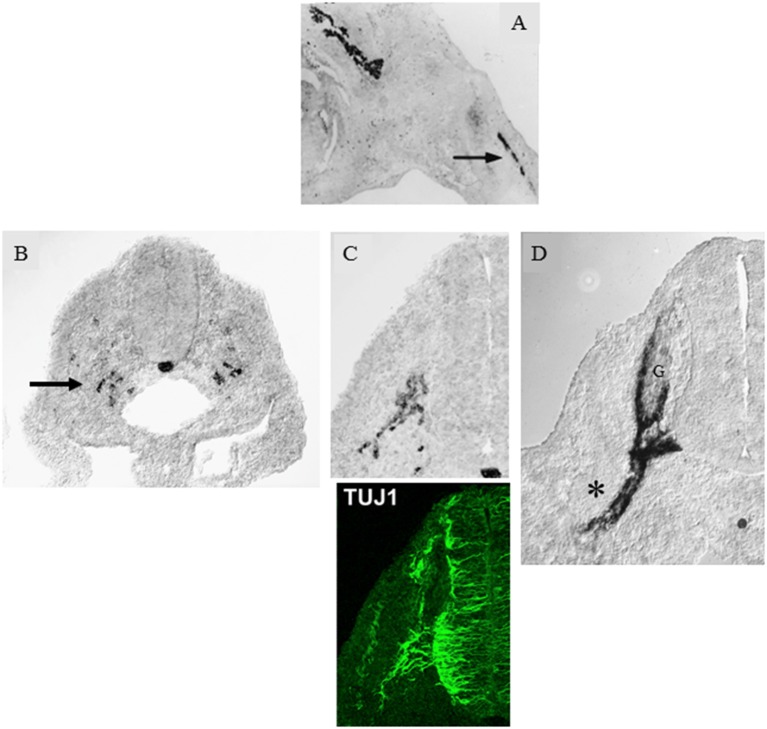
*Myelin protein zero (mpz)* gene expression at the onset of Schwann cell development. **(A)** *In situ* hybridization showing *mpz* expression in iSch of embryonic (E16) rat nerves (nerve in developing limb arrowed). **(B)** *In situ* hybridization showing an initial appearance of *mpz* expression in scattered neural crest cells at the level with the ventral developing spinal cord (e.g., arrow). Strong *mpz* labeling is also seen in the notochord. Transverse section from an E9/10 rat embryo. **(C)** When axons emerge from the ventral spinal cord (shown by TuJ1 immunolabeling in the lower panel), *mpz* expressing cells cluster nearby (upper panel). Transverse section from an E10/11 rat embryo. **(D)** In E12/13 rat embryo, *mpz* expression is seen in nascent spinal nerves (asterisk), and in the ventral root and ventral and lateral aspects of the dorsal root ganglion (G) (with permission, modified from Lee et al., 1997).

In adult nerves, the *myelin protein zero (mpz)* gene which encodes the major myelin protein zero (P0), is strongly expressed in myelin Schwann cells and restricted to these cells. But after nerve injury, the gene is rapidly activated to achieve a low, basal level of expression in Remak cells as they loose axonal contact. Similarly during development, low, basal levels of *mpz* gene expression are seen long before myelination. It is readily detected in iSch and SCP and is seen in a subpopulation of neural crest cells even before axonal outgrowth from the ventral part of the developing spinal cord (Figure 2; Bhattacharyya et al., 1991; Lee et al., 1997, 2001). As the first axons extend into the mesenchyme, *mpz* expressing cells accumulate nearby, and soon afterward they integrate with the axons to form a compact early spinal nerve.

**Figure 3:**
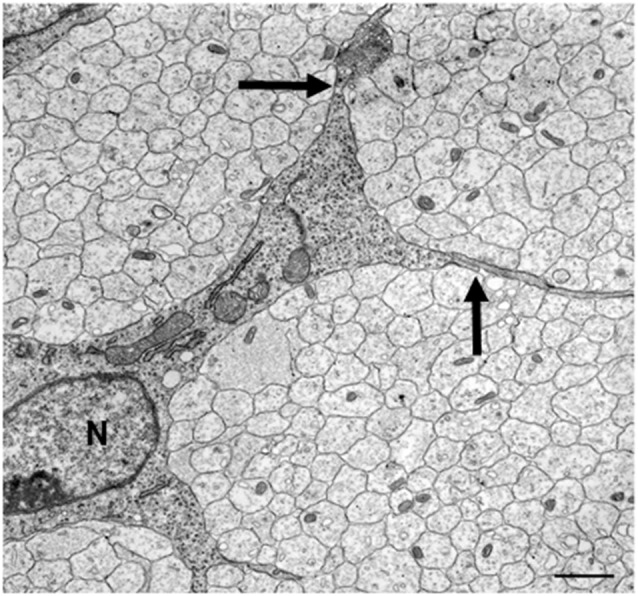
The tight association between SCP and axons in developing peripheral nerves. This electron micrograph shows a transverse section through the sciatic nerve of an E14 rat embryo. Note the intimate association between large groups of axons and SCP, and the absence of significant extracellular spaces and connective tissue. Parts of three SCP are visible. Part of the nucleus (N) and the cell body of one of them is included in the field. Arrows point to the junctions between this cell and processes from two other SCP. Scale bar: 1.7 μm (with permission from Jessen and Mirsky, 2005b).

**Figure 4:**
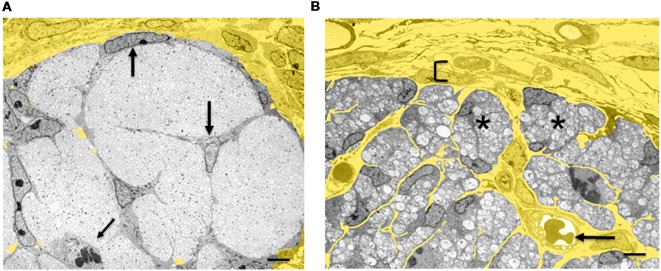
Architectural reorganization of developing nerves. **(A)** Electron micrograph of a transverse section through E14 rat sciatic nerve. SCP are embedded among the axons and at the surface of the nerve (big arrows). A dividing SCP is also seen (small tilted arrow). Note that connective tissue (yellow) surrounds the nerve, but is not found inside the nerve. **(B)** Electron micrograph of a transverse section through E18 rat sciatic nerve. iSch surround the collection of axons, forming compact groups (“families”; examples indicated by asterisks). Extensive connective tissue (yellow) containing blood vessels (arrow) is now found inside the nerve surrounding the families, as well as outside the nerve. Bracket indicates the developing perineurium (scale bar: 4 μm).

Electron microscopy of these nerves shows that these cells, SCP, are intimately associated with large bundles of axons, which they envelop communally with flattened membranous processes (Figures 3, 4A). In larger nerve branches, the cells embed themselves deep among the axons, and they are also tightly associated with axons at the nerve surface, both in small and large nerve branches (Jessen and Mirsky, 2005a; Wanner et al., 2006b).

**Figure 5:**
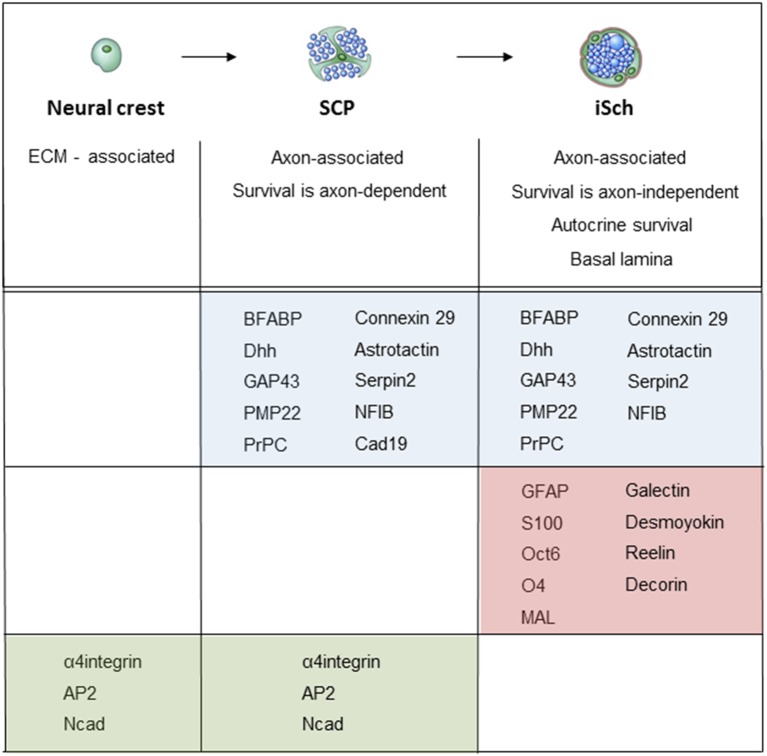
Molecular expression at the main stages of the embryonic Schwann cell lineage. Below the lineage drawing, main cell biological differences between each developmental each stage are indicated. The molecular markers of each stage divide into three groups: (i) markers that are upregulated at the crest/SCP transition, and maintained by iSCh (blue); (ii) markers that are upregulated at the SCP/iSch transition (pink); and (iii) markers that present on neural crest cells and SCP but are downregulated at the SCP/iSch transition (green). For references, see Jessen and Mirsky (2005a) where more detailed comments on gene expression patterns are provided.

When neural crest cells that migrate in the mesenchyme convert to SCP that reside within nerves, they change the expression of several hundred genes (Buchstaller et al., 2004). Two to three-fold higher number of genes are up-regulated than down-regulated, and up-regulated genes include a broad spectrum of functional classes, such as transcription factors, cell cycle regulators, cytoskeleton-associated molecules and molecules involved in cell growth and differentiation. Large number of these genes, such as the genes encoding for MBP, PLP22, desert hedgehog (Dhh) and BFABP, are Schwann cell-associated in the sense that their expression persists or is further up-regulated during subsequent Schwann cell development (Figure 5; Jaegle et al., 2003; Buchstaller et al., 2004; Takahashi and Osumi, 2005; D’Antonio et al., 2006b; Li et al., 2007).

The SCP described here are glial cells for the following reasons. First, the tight and intimate association with neurons and their processes, and envelopment of axons is a defining feature of glial cells in the normal PNS and CNS, and this holds true both for development and the adult state. Second, the transition from crest cells to SCP involves large-scale changes in gene expression, and among the genes that are activated in SCP are many glial-associated genes that are also expressed by Schwann cells (Figure 5). Third, SCP, but not crest cells, are strikingly dependent on axonal signals for survival and differentiation. This is a characteristic feature of Schwann cells, where axonal signals are essential for proliferation and differentiation. Neuregulin 1 (NRG1) type III is a major axonal signal controlling both SCP survival and Schwann cell differentiation. Accordingly, in NRG1 mutants, SCP and iSch are depleted from peripheral nerves although neural crest cells survive and generate crest derivatives such as dorsal root ganglia (DRG) neurons and satellite cells (Birchmeier and Nave, 2008; Birchmeier, 2009).

In addition to the above, other difference between crest cells and SCP is their contrasting response to potential survival factors, including IGF1, endothelin, PDGF and NT3 and cell substrates such as laminin and fibronectin (Woodhoo et al., 2004). Further, in comparison to neural crest cells, SCP are not sensitive to the neurogenic effect BMP2 and are biased towards the generation of iSch (Kubu et al., 2002; Woodhoo and Sommer, 2008).

## Early Glial Cells in the PNS and CNS Are Multipotent

The SCP in newly formed embryonic nerves represent the earliest well defined glial-like cell in the development of the PNS. In CNS development, the best-studied early glial cell is the radial glial cell, a cell that conforms to the phenotype of glial cells on the basis of ultrastructural, molecular and cell biological criteria, much like SCP (Pinto and Götz, 2007). Radial glia have now been shown to be multipotent and to directly or indirectly generate neurons and oligodendrocytes, although these cells were initially thought to generate only astrocytes (Malatesta and Götz, 2013). It is therefore not surprising that SCP share this feature of multipotency, and can give rise to other cell types during development in addition to iSch.

Cell culture studies first showed that SCP were not restricted to forming Schwann cells only (Ciment et al., 1986; Morrison et al., 1999; Dupin et al., 2000). The first demonstration of this *in vivo* was a study of fibroblast generation using Cre-lox-based based lineage tracing in mice (Joseph et al., 2004). Unlike the CNS, peripheral nerves contain a substantial amount of connective tissue and therefore fibroblasts (5%–10% of the number of Schwann cells at birth; Jessen and Mirsky, 2005a). These cells could be traced to cells in the nerve that express Dhh, a gene that is expressed in SCP and iSch but not in the neural crest, and which controls the correct formation of the perineurial nerve sheath (Parmantier et al., 1999; Jaegle et al., 2003; Joseph et al., 2004; Sharghi-Namini et al., 2006). The finding that SCP convert to both iSch and fibroblasts is in good agreement with the observation that in developing nerves the appearance of iSch and fibroblasts is temporarily linked with the disappearance of SCP (Wanner et al., 2006a).

There is also *in vivo* evidence that some neurons in the enteric nervous system of the gut wall arise from SCP (Uesaka et al., 2015; Espinosa-Medina et al., 2017), and the same applies to neurons in parasympathetic ganglia (Dyachuk et al., 2014; Espinosa-Medina et al., 2017). In the case of parasympathetic neurons, there is evidence that SCP that express Phox2b preferentially generate neurons, while this factor is downregulated in SCP that generate iSch. SCP also give rise to a significant number of adrenal chromaffin cells, activating Phox2b and Ascl1 expression as they migrate from embryonic nerves (Furlan et al., 2017). Similarly, some SCP in distal nerves escape NRG1 signaling from axons as they generate melanoblasts and emigrate to take up appropriate location in the nearby developing skin and develop to melanocytes, a process that requires downregulation of the transcription factor Foxd3 and cross-regulatory interactions between the transcription factors Sox2 and Mitf (Adameyko et al., 2009, 2012; Nitzan et al., 2013). Lastly, mesenchymal stem cells in the teeth that produce odontoblasts and tooth pulp cells can originate in SCP (Kaukua et al., 2014; Kalcheim and Kumar, 2017).

Thus, both radial glia and SCP show the stem cell-like feature of multipotency, although it cannot be excluded that *in vivo* some SCP are already lineage-restricted (Kalcheim and Kumar, 2017). This interesting property of developing CNS and PNS glial cells relates differently to lineage progression in the two systems. In the case of SCP, broad developmental potential represents the retention of a notable feature of their cell of origin, neural crest cells, while in radial glia, multipotency appears during development as a component of the radial glial phenotype.

The capacity of cells with a glial phenotype to generate more than one cell type can be retained even in the adult. This is seen in the case of subventricular zone astrocytes, which give rise to neurons and oligodendrocytes in the mature brain (Kriegstein and Alvarez-Buylla, 2009).

## The Plasticity of the Schwann Cell Phenotype

Another example of the ability of glial cells to convert to different phenotypes is seen in adult nerves, where Schwann cells retain the capacity to alternate between radically different Schwann cell phenotypes in response to cell-extrinsic signals. Thus, it is well established that Remak and myelin cells, although strikingly different in structure, molecular expression and function, are potentially interchangeable, the two phenotypes being exclusively determined by signals from the axons they associate with. A further instance is the capacity of myelin and Remak cells to give rise to a third adult Schwann cell phenotype, the repair Schwann cell, in response to the different signaling environment generated by nerve injury (Arthur-Farraj et al., 2012, 2017; Jessen et al., 2015a; Jessen and Mirsky, 2016, 2019; Gomez-Sanchez et al., 2017). The strong regenerative potential of peripheral nerves (Chen et al., 2007; Brosius Lutz and Barres, 2014; Cattin and Lloyd, 2016; Jessen and Mirsky, 2019) is to a significant extent due to this adaptive injury response, which reprograms myelin and Remak cells to a Schwann cell that is specialized in morphology and molecular expression to support repair. Repair Schwan cells engineer the clearance of myelin, upregulate expression of trophic factors and cell surface molecules that prevent the death of inured neurons and stimulate axon regrowth. These cells also elongate and branch to form compact cellular columns, classically termed Bungner bands, along which axons regenerate back to their targets (Gomez-Sanchez et al., 2017). When axons have regenerated, signals from small and large axons convert repair cells back to the phenotype of Remak and myelin cells, respectively, thereby restoring normal nerve function. Lastly, Schwann cell plasticity is also demonstrated by the pigmentation of injured nerves, since Schwann cells are the most likely cell of origin of the melanocytes that appear around nerve fascicles after damage, although the lineage of these cells has not been directly determined (Rizvi et al., 2002).

## The Generation of Glial Cells by the Neural Crest: The Appearance of Schwann Cell Precursors

Much remains to be learned about the molecular signaling that directs crest cells to enter peripheral nerves, transform to cells with glial phenotype, and form and intimate association with axons (Lobsiger et al., 2002; Woodhoo and Sommer, 2008; Jacob, 2017; Kalcheim and Kumar, 2017). Although neither transcription factors nor cell-extrinsic signals that uniquely specify these events have been identified, recent work has revealed that histone deacetylase 1/2 (HDAC1/2) and the transcription factor Sox10 have an important role in this process. This will be outlined in the subsequent sections, together with earlier studies on NRG1 and Notch.

### Histone Deacetylase (HDAC) 1/2

Enzymes and complexes controlling chromatin remodeling take part in governing the development of Schwann cells in embryonic nerves and postnatal myelination. After an injury, they also influence the generation of the repair Schwann cell phenotype and the re-myelination (Jacob, 2017; Ma and Svaren, 2018). HDAC1/2 control the formation of SCP from crest cells by combining with the Sox10 to activate Pax3, a protein that has long been implicated in early Schwann cell development (Grim et al., 1992; Doddrell et al., 2012; Jacob et al., 2014). Together Sox10 and Pax3 activate the MSCS4 Sox10 enhancer to promote the crest/SCP transition (Wahlbuhl et al., 2012). A complex involving Pax3, Sox10 and HDAC1/2 activates the *mpz* promoter and thereby initiates a low-level expression of P0 protein and mRNA, which is a marker of early development of glial cells from the crest as mentioned in the previous section “Schwann Cell Precursors Show an Obvious Glial-Like Phenotype”; (Lee et al., 1997). Sox10 also interacts with the *fabp7* promoter to initiate expression of BFABP, and other markers of the crest/SCP transition (Jessen and Mirsky, 2005a; Jacob, 2017).

### Sox10

Sox10 has an extensive involvement in Schwann cell development, from the emergence of the lineage to the regulation of the transcription factors Oct6 and Krox20 (Egr2) that control myelination (Schreiner et al., 2007; Svaren and Meijer, 2008; Finzsch et al., 2010). In line with this, Sox10 potentially functions to maintain expression of ErbB3 receptors for NRG1, a signal with a broad role in the Schwann cell lineage (Britsch et al., 2001). In neural crest cells, there is evidence that Sox10 is also important for maintenance of developmental multipotency in combination with other factors such as Notch (Kim et al., 2003; Wahlbuhl et al., 2012). In mice with genetic inactivation of Sox10, the crest initially generates neurons in DRG normally. However, in the absence of Sox10, the emergence of the glial component fails, since both satellite glial cells in the ganglia, and SCP in peripheral nerves are missing (Britsch et al., 2001; Sonnenberg-Riethmacher et al., 2001; Wahlbuhl et al., 2012). Nevertheless, Sox10 does not uniquely specify PNS glia, because this protein is found in essentially all neural crest cells and initially in developing melanocytes.

### NRG1

NRG1 is broadly involved in controlling Schwann cell biology (Birchmeier and Nave, 2008; Birchmeier, 2009; Monk et al., 2015). But there are a number of reasons why this factor is unlikely to be required for the generation of glial cells from the neural crest. First, Schwann cells are generated in spite of inactivating mutations in the NRG1 receptor ErbB3 in zebrafish (Lyons et al., 2005). Second, a major category of PNS glia closely related to Schwann cells, satellite cells in DRG ganglia, appear to been normally generated in mice in which functional NRG1 or the NRG1 receptors ErbB2 and ErbB3 are missing. Third, cells expressing the SCP marker *mpz* mRNA are normally generated in rat neural crest cultures without the addition of NRG1 (Lee et al., 1997; Woodhoo et al., 2009). GFAP positive Schwann cells also emerge readily in neural crest without NRG1 (Shah et al., 1994). Exposure to NRG1 increases the number of GFAP positive cells in such cultures, an effect likely to reflect the well-established role of NRG1 as a survival factor, mitogen and lineage progression factor for SCP, functions that will be discussed in a later section. All of this argues that NRG1 is unlikely to be required for the conversion of crest cells to glial cells.

Indirectly, NRG1 may, however, boost gliogenesis, since in crest cultures NRG1 is a potent inhibitor of neurogenesis. It can be speculated that this would increase the time during which crest cells are exposed to gliogenic signals and therefore promote gliogenesis (Shah et al., 1994).

### Notch

During CNS development, Notch supports the generation of glial cells (Kubu et al., 2002; Yoon and Gaiano, 2005; Louvi and Artavanis-Tsakonas, 2006). The number of GFAP positive Schwann cells that appear in neural crest cultures is increased by Notch activation, GFAP, however, is not a maker of SCP generation, but of the later event of iSch appearance from SCP (Figure 5), and this effect has not been observed in studies on crest cells from mouse and chick that use the earlier differentiation marker *mpz* mRNA, which detects SCP (Wakamatsu et al., 2000; Woodhoo et al., 2009). Also in ovo, Notch activation did not stimulate Schwann cell generation (Morrison et al., 2000). In mice lacking a key mediator of Notch signaling, the transcription factor RBPJ, Schwann cells were generated normally, although the emergence of satellite cells and neurons were impaired (Taylor et al., 2007). Similarly, the appearance of SCP was unaffected in mice lacking Hes1 and Hes5, important effectors of canonical Notch signaling (Woodhoo et al., 2009). These observations argue against Notch as a significant regulator of the appearance of SCP in spinal nerves. The increase in GFAP positive Schwann cells in crest cultures exposed to Notch activation is likely explained by the strong mitogenic effects of Notch on iSch that appear in these cultures under the influence of other signals (see “Notch” section).

## NRG1 and Notch in Schwann Cell Precursors

In mice, inactivating mutations in NG1 or the NRG1 receptors ErbB2 and ErbB3 have a dramatic effect on SCP, which are radically depleted or absent from embryonic nerves (Birchmeier and Nave, 2008; Birchmeier, 2009; Taveggia et al., 2010; Monk et al., 2015). Because NRG1 is unlikely to be needed for SCP generation (see “NRG1” section), this phenotype points to the importance of NRG1 for the survival and migration of SCP.

### Survival

If SCP are dissociated from nerves of E14 embryos (E12 in the mouse) and placed in a culture, they undergo apoptotic death within 12–14 h (Jessen et al., 1994). This contrasts with iSch even from only 3-day old embryonic nerves, and with Schwann cells from newborn nerves, since these cells can readily be maintained in routine culture media (Dong et al., 1995). *In vitro*, SCP death can be prevented by co-culturing the cells with neurons or by culturing them in a neuron-conditioned medium. The active component in the neuron-conditioned medium has been identified as NRG, and exogenously applied NRG1 prevents SCP death (Dong et al., 1995). *In vivo*, axonal degeneration also triggers the death of SCP, which is prevented by exposure to NRG1 (Winseck and Oppenheim, 2006). In mouse embryos, NRG1 is found on axonal surfaces (Taveggia et al., 2005), and NRG1 is expressed by DRG and motor neurons at the stage when SCP are present in peripheral nerves. This factor is therefore expressed at the right place and time to function as a trophic signal that blocks SCP apoptosis (Marchionni et al., 1993). All of this suggests that NRG1 on embryonic axons is a key regulator of SCP survival, and thereby indirectly of Schwann cell generation. This is likely to be a major reason for the depletion for SCP from embryonic nerves in NRG and NRG receptor mutants.

### Migration

The migration of the trunk neural crest cells is adversely affected in NRG mouse mutants in which NRG1 signaling is inactivated (Britsch et al., 1998). Although the cells migrate apparently normally to the sites of DRG formation, they fail to reach the more distant sites where sympathetic ganglia are generated. In zebrafish nerves, NRG1 stimulates the migration of developing Schwann cells, and in culture, NRG1 enhances migratory activity of SCP and Schwann cells (Meintanis et al., 2001; Lyons et al., 2005; Yamauchi et al., 2008; Raphael and Talbot, 2011; Perlin et al., 2011; Miyamoto et al., 2017). From this, it is likely that impaired migration contributes to the dramatic reduction on SCP number in mouse NRG1 mutants.

### The Presentation of NRG1 on Developing Axons

The main NRG1 isoform on axonal surfaces is isoform III, which therefore mediates most of the effects of NRG1 on SCP and iSch. Because this protein has two transmembrane domains, proteolytic cleavage is required to effectively present the active EGF domain on the axonal surface, allowing it to bind in a juxtacrine fashion to ErbB2/3 receptors on a nearby glial cell (Taveggia et al., 2005; Birchmeier, 2009). Proteolysis to activate NRG1 is carried out by the membrane-associated protease BACE1 (beta secretase). Another protease which also acts on NRG isoform lll and affects myelination is TACE (ADAM17; Hu et al., 2006; Willem et al., 2006; La Marca et al., 2011). It is of interest that a cytoplasmic domain of axonal NRG1 can engage in back-signaling and communicate with the neuronal cell body to stimulate prostaglandin synthesis and myelination (Bao et al., 2003; Trimarco et al., 2014).

### Amplification of NRG1 Signaling by Notch

In addition to NRG1, developing axons also express Notch ligands, while Notch is found on SCP and iSch. Although axonal Notch-SCP signaling does not directly support SCP survival, it amplifies the capacity of NRG1 to do so (Woodhoo et al., 2009). This is likely explained by the fact that Notch activation increases the expression of ErbB2 NRG1 receptors on SCP. This effect is seen when Notch signaling is activated in cultured Schwann cells and is confirmed *in vivo*, because reduced ErbB2 levels in E14 nerves are found in mice with conditional inactivation of Notch signaling in SCP and iSch (Woodhoo et al., 2009). The importance of axonal Notch signaling for sustaining ErbB2 levels and NRG1 signaling from axons to developing Schwann cells is likely to explain another effect of Notch on SCP. This is the acceleration of the conversion of SCP to iSch (Woodhoo et al., 2009). This is seen *in vivo*, in mice with conditional inactivation of RBPJ, which transduces canonical Notch signaling, since these mice show delayed appearance of iSch. Confirming this role of Notch, iSch appear prematurely in mice where Notch is overexpressed (Woodhoo et al., 2009). Together these data indicate that Notch acts as a positive regulator of NRG1 signaling in embryonic Schwann cell development, thereby indirectly controlling SCP survival and lineage progression.

## A Comparison of Immature Schwann Cells and Schwann Cell Precursors

iSch appear in mouse nerves at E15/16 (E 17/18 in rat). The main phenotypic differences between SCP and iSch include the following. First, iSch, but not SCP, survive without exposure to axonal survival signals and suppress apoptosis by autocrine survival loops (Jessen et al., 1994; Dong et al., 1995; Meier et al., 1999). Second, iSch and SCP differ substantially in gene expression (Buchstaller et al., 2004; D’Antonio et al., 2006b). Among a large number of genes that are up-regulated at this transition are S100 and GFAP, which are frequently used markers of iSch, while down-regulated genes include the transcription factor AP2, α4 integrin, and Ncadherin. Cadherin19 is up-regulated at the neural crest/SCP transition but down-regulated at the SCP/iSch transition. It is so far the only gene known to be expressed selectively in SCP only. Third, iSch, but not SCP develop a basal lamina (Wanner et al., 2006a). Fourth, under standard survival conditions *in vitro*, SCP migrate at about 10-fold the rate of iSch (Jessen et al., 1994).

Perhaps the most striking difference between SCP and iSch is in the way their survival is controlled. As mentioned previously, a population of SCP from E14 (rat; E12 mouse) nerves placed in culture without neurons dies completely and rapidly, and cannot be rescued by increasing cell density. This true irrespective of whether the cells are plated on artificial or extracellular matrix substrate or exposed to defined or serum-containing medium. In contrast, iSch from E17/18 nerves survive under these conditions, although their survival is markedly density dependent, a classical indicator of autocrine survival support (Jessen et al., 1994; Dong et al., 1995, 1999; Gavrilovic et al., 1995; Meier et al., 1999). The autocrine survival signal consists of a number of factors, including IGF2, NT3, PDGF-B, leukemia inhibitory factor, and lysophosphatidic acid (Dowsing et al., 1999; Meier et al., 1999; Weiner and Chun, 1999).

## Signals That Control the Generation of Immature Schwann Cells From Schwann Cell Precursors

While several signals accelerate or decelerate the generation of iSch (is *in vivo* as outlined below), it is notable that exposure of essentially pure cultures of SCP to NRG1 alone in defined medium results in the conversion of the SCP to iSch on schedule. On the basis of a number of criteria, including the appearance of iSch markers, morphology and appearance of autocrine survival loops, essentially the whole population of E14 rat SCP converts to cells with the phenotype if E18 iSch during 4 days, matching the time course of Schwann cell generation *in vivo* (Dong et al., 1995).

The role of Notch as an appositive regulator of the SCP/iSch transition was discussed earlier (see “NRG1 and Notch in Schwann Cell Precursors” section). Another factor involved in lineage progression is Sox10. This transcription factor is expressed in neural crest cells and in the Schwann cell lineage throughout development and in the adult. At the SCP/iSch transition, dimeric Sox10 is recruited to the *Oct6* Schwann cell enhancer (SCE) which promotes expression of the transcription factor Oct6 in iSch. Subsequently Oct6, with Sox10 and Brn2, are recruited to the *Krox20* myelin Schwann cell enhancer (MSE) to increase expression of the pro-myelin transcription factor Krox20 and permit myelination (Topilko et al., 1994; Bermingham et al., 1996; Jaegle et al., 1996; Ghislain and Charnay, 2006; He et al., 2010; Jagalur et al., 2011).

Negative regulation of the appearance of iSch is carried out by endothelin. Endothelin delays the generation of iSch from SCP in culture, and in agreement with this inactivation of endothelin B receptors *in vivo* causes a premature appearance of iSch (Brennan et al., 2000). Endothelin B receptor mRNA is suppressed by the transcription factor Zeb2, which also has a role in controlling factors that delay myelination (Quintes et al., 2016; Wu et al., 2016). Another negative regulator is the transcription factor AP2. In cultured SCP, enforced expression of AP2 retards the appearance of iSch, and, as might be expected of a negative regulator of this transition, AP2 is strongly down-regulated as SCP convert to iSch *in vivo* (Stewart et al., 2001).

## Developing Schwann Cells Organize the Protective Tissues of the PNS

While the CNS is surrounded by the hard connective tissue of the skull and vertebrae for protection, the PNS is supported from within, by the flexible collagen-rich extracellular matrix of the endoneurial connective tissue that surrounds individual axon-Schwann cell units. Additionally, nerves are surrounded by a multi-layered cellular tube, the perineurium, and outside of that, the collagenous layer of the epineurium. SCP and iSch play a key role in organizing both these nerve-intrinsic and nerve- extrinsic protective systems.

### The Architectural Transformation of Nerve: The Generation of Connective Tissue

The SCP/iSch transition coincides with a radical reorganization of the relationship between nervous tissue and connective tissue (Wanner et al., 2006a). At the SCP stage, connective tissue is excluded from nerves and is restricted to the nerve surround (Figures 3, 4A). These nerves, therefore, consist exclusively of neurons (axons) and glia. As SCP progress to generate iSch they also give rise to fibroblasts that remain within nerves (see “Early Glial Cells in the PNS and CNS Are Multipotent” section). Fibroblasts secrete extracellular matrix molecules, especially collagen, which come to occupy the extracellular spaces that open up within the nerve at the SCP/iSch transition. At the same time, this endoneurial connective tissue becomes vascularized and basal lamina is formed around iSch. Thus, from the iSch stage (E18 in rat) onwards, peripheral nerves are a mosaic tissue, consisting of axon-Schwann cell bundles (“families” described in newborn nerves; Peters and Muir, 1959; Webster and Favilla, 1984) intermixed with vascularized connective tissue (Figure 4B).

The signaling that controls whether SCP generate fibroblasts or iSch are not known, and the molecular mechanisms that determine the accompanying changes in cell and tissue relationships are also unclear, although it has been suggested that they are related to a strong reduction in the expression of N-cadherin by SCP and iSch that takes place at this time (Wanner et al., 2006a).

### Signaling Between Glial Cells and Connective Tissue Directs the Formation of the Perineurium and Epineurium

The perineurial sheath serves as a nerve-tissue barrier that protects nerves from unwanted cells and molecules. The cells of this sheath are covered by a basal lamina and joined by desmosomes and tight junctions. Although these features, and the barrier function of the perineurium are typical of epithelia, the perineurium appears to be derived from connective tissue (Bunge et al., 1989). The nascent perineurium can first be discerned in the connective tissue surrounding developing nerves at the iSch stage in the embryo The further development of this structure, requires signaling from developing Schwann cells mediated by Schwann cell Dhh binding to Patch receptors in the surrounding tissue (Parmantier et al., 1999). In mice with genetic inactivation of the *Dhh* gene, both the perineurium and the surrounding collagen layer of the epineurium fail to develop properly, and mini-fascicles made by perineurial-like cells develop within the endoneurium. This results in failure of the nerve-tissue barrier and a broad-spectrum nerve pathology, both in the mice, and in humans with inactivating *Dhh* mutations (Parmantier et al., 1999; Umehara et al., 2000).

## The Function of Schwann Cell Precursors and Immature Schwann Cells

During development, SCP act as a developmental hub, generating iSch but also diversifying to make contributions to several cell populations that also originate from the neural crest along other routes (see “Early Glial Cells in the PNS and CNS Are Multipotent” section). iSch, however, have narrower developmental options and serve as a pool from which the Remak or myelin Schwann cell phenotypes are induced by cell-extrinsic signaling associated with axons and the basal lamina. In addition to playing these roles in lineage progression, SCP and iSch have been implicated in the control of neuronal survival, nerve fasciculation and the formation of the neuro-muscular junction.

### Neuronal Survival

Glial cells have long been implicated in the regulation of neuronal survival, both during development and in the adult. One of the more compelling observations in this regard comes from experiments on mice with inactivating mutations in *Sox10* or components of the NRG1 signaling pathway (Riethmacher et al., 1997; Woldeyesus et al., 1999; Garratt et al., 2000a; Wolpowitz et al., 2000; Britsch et al., 2001). Because of the importance of these molecules for Schwann cell development, early PNS glia cells are missing or found in severely reduced numbers in these mutants.

Perhaps surprisingly, these mice reveal that early glia are not important for the initial differentiation of DRG neurons. Moreover, embryonic nerves containing the axons of motor and DRG neurons extend towards their target areas broadly following the normal pattern of nerve outgrowth without early glia. The unexpected irrelevance of early PNS glia for nerve pathfinding and outgrowth pattern is confirmed in the *Splotch (Pax3)* mouse mutant, where booth SCP and DRG are missing, but nerve growth pattern remains broadly normal, and by observations on the initial outgrowth of the lateral line in zebrafish (Grim et al., 1992; Gilmour et al., 2002; Raphael and Talbot, 2011).

On the other hand, in spite of the fact that without PNS glia motor and DRG neurons are generated normally in Sox10 or NRG1 pathway mutants, later in embryonic development, there is a dramatic loss of both these neuronal populations, especially at the limb levels (Woldeyesus et al., 1999). This suggests that after neurogenesis, SCP, iSch and perhaps also satellite cells in ganglia have an important role in sustaining early DRG and motor neurons. It is intriguing that because SCP survival depends on neurons (see “Survival” section) neurons and glia in embryonic nerves appear to depend on each other for survival.

### Nerve Fasciculation and the Formation of the Neuromuscular Junction

Although the initial pattern of nerve outgrowth towards target tissues is relatively normal without early PNS glia, within these tissues nerve branching is aberrant, and without SCP nerves are poorly fasciculated and disorganized (Woldeyesus et al., 1999; Wolpowitz et al., 2000).

Similarly, early stages of synapse formation proceed without accompanying glial cells, including the appearance of some of the ultrastructural features of the neuromuscular junction and acetylcholine receptor clustering. Postsynaptic junctional folds fail to form normally, however, and there are abnormal terminal sprouting and erroneous location of synaptic sites (Morris et al., 1999; Woldeyesus et al., 1999; Lin et al., 2000; Wolpowitz et al., 2000).

In view of the cellular architecture of growing nerve endings, it is not surprising that nerve terminals fail to behave normally within target tissues in the absence of accompanying glial cells (Wanner et al., 2006a). SCP are abundant at the nerve front, where ultrastructural analysis shows that they generate complex scaffolds around axonal growth cones. Notwithstanding the apparently irregular and disorganized aspect of these structures, a quantitative ultrastructural analysis reveals that the amount of membrane contact is remarkably constant from one nerve front to another (Wanner et al., 2006b). This cellular arrangement is clearly essential for axons to establish normal connections with their targets.

## Signals That Match the Numbers of Schwann Cells to the Number of Axons

There is evidence that Schwann cell numbers are controlled both by signals that induce Schwann cell death and by mitogens associated with axons, the basal lamina that starts to form at the iSch stage, and perhaps from other sources within the nerve.

### Signals Regulating Survival and Death

Two main factors have been implicated in actively killing Schwann cells, transforming growth factorβ (TGFβ), acting through TGFβ type II receptors, and nerve growth factor (NGF) acting through p75NTR.

TGFβ type II receptors mediate normal developmental death of Schwann cells in perinatal nerves, presumably functioning together with the mitogens discussed earlier to achieve correct cell numbers (D’Antonio et al., 2006a). Although most Schwann cell survives without axons (see “Survival” section) nerve injury, especially in neonates, it results in a transient phase of increased apoptosis (Grinspan et al., 1996; D’Antonio et al., 2006a). This injury-induced death is also in part mediated by TGFβ acting on TGFβ type II receptors (D’Antonio et al., 2006a).

NGF acting through p75NTR does not appear to take part in the control of survival during development, although NGF contributes together with TGFβ to the Schwann cell death in injured nerves (Syroid et al., 2000).

HDAC1/2 is also involved in Schwann cell survival pathways, since substantial cell death is seen from postnatal day 2 onwards in mice in which HDAC1/2 has to be inactivated at the SCP/iSch stage (Jacob et al., 2011).

Laminin, a major component of the basal lamina, can promote Schwann cell survival *in vitro* independently of the potential function of laminin as a mitogen discussed below (see “Nerve Fasciculation and the Formation of the Neuromuscular Junction” section; Meier et al., 1999). Other studies confirm that plating Schwann cells on laminin results in increased cell numbers, but have not determined whether this effect is due to the promotion of survival or proliferation (McGarvey et al., 1984; Yang et al., 2005; Yu et al., 2005, 2009). *In vivo*, Schwann cell death is also increased after genetic inactivation of laminin in Schwann cells (Yu et al., 2005; Chernousov et al., 2008; Feltri et al., 2016).

A large number of soluble signals, including NRG1, promote Schwann cell survival *in vitro* or when exogenously applied to Schwann cells *in vivo* (reviewed in Jessen and Mirsky, 2013).

### Mitogens

*In vivo*, BrdU incorporation indicating DNA synthesis is seen in SCP, and at gradually increasing levels in iSch to reach a peak just before birth (Stewart et al., 1993). After birth, DNA synthesis declines rapidly as iSch are induced to assume first the myelin and then the Remak phenotypes (Stewart et al., 1993). Evidence that axons drive Schwann cell proliferation comes from the observation that nerve transection in neonatal rodents, which leads to axonal degeneration, results in the decline in proliferation (Komiyama and Suzuki, 1992), and from the localization of potential mitogens such as Notch ligands and NRG1 on axonal membranes. Similarly, *in vitro* contact between axons and Schwann cells leads to Schwann cell proliferation (Salzer and Bunge, 1980).

NRG1 is commonly implicated as a significant component of the mitogenic signal between axons and Schwann cells. In support, exposure to soluble NRG1 drives cell division in cultured Schwann cells, and in co-cultures, the mitogenic effect of axons on Schwann cells is mediated by NRG1 (Morrissey et al., 1995). Also, in zebrafish blockers of ErbB2 NRG1 receptors reduce Schwann cell proliferation. In rodents, however, direct evidence for the plausible notion that NRG1 is an axon-associated mitogen is still missing.

There are also intriguing contrary findings. Thus, conditional inactivation of ErbB2 NRG1 receptors in perinatal nerves results in increased Schwann cell proliferation (Garratt et al., 2000b). While this may be related to impairments in myelination seen in these mice, the mitogen driving Schwann cell division in these nerves is unlikely to be NRG1. In cyclin D1^−/−^ mice, Schwann cells also divide normally, although cell culture experiments indicate that cyclin D1 is required for the mitogenic effect of NRG1 (Garratt et al., 2000a, b; Kim et al., 2000; Atanasoski et al., 2001; Finzsch et al., 2010). Lastly, in *Sox10* mutants, mRNA levels for the NRG1 receptor ErbB3 are substantially reduced in Schwann cells although Schwann cell proliferation is unchanged or increased (Finzsch et al., 2010). Injury of adult nerves results in a transient wave of Schwann cell proliferation, which also appears to be independent of ErBbB2 NRG1 receptors (Atanasoski et al., 2001, 2006).

In addition to axons, signals that drive Schwann cell proliferation during development also come from sources. This includes laminin in the basal lamina since genetic deletion of Schwann cell laminin (γ1 chain mutants or dy2J/α double mutants) results in a drop in proliferation rate. Other basal lamina-associated factors that promote Schwann cell proliferation *in vivo* include the small Rho GTPase cdc42 and focal adhesion kinase (FAK; Yu et al., 2005; Grove et al., 2007; Chernousov et al., 2008; Feltri et al., 2008, 2016). Jun activation domain-binding protein (Jab1) has been suggested to mediate signaling downstream of laminin 211, and genetic inactivation of Jab1 in Schwann cells results in reduced proliferation (Porello et al., 2014). At least four other signaling pathways have been shown to be involved in driving Schwann proliferation in developing nerves *in vivo*, namely Notch, TGFβ, and the Hippo and cAMP pathways.

#### Notch

Notch signaling is a major axonal driver of Schwann cell proliferation in developing nerves. *In vivo* genetic inactivation of Notch selectively in Schwann cells results in about 50% reduction in Schwann cell DNA synthesis in developing nerves, and this is accompanied by a reduction in Schwann cell numbers (Woodhoo et al., 2009). Notch ligands are also expressed on peripheral axons. Further, *in vitro* Notch activation in Schwann cells is strongly mitogenic (Woodhoo et al., 2009).

#### TGFβ

Potentially, TGFβ has two distinct effects on Schwann cells, induction of death as discussed above (see “Signals Regulating Survival and Death” section), and induction of DNA synthesis. Both effects can be seen *in vitro* depending on culture conditions (Einheber et al., 1995; Parkinson et al., 2001). Both effects can also be seen *in vivo* (D’Antonio et al., 2006a). When TGFβ type II receptor is genetically inactivated selectively in Schwann cells to abolish TGFβ signaling, Schwann cell DNA synthesis in developing nerves is substantially reduced, showing that TGFβ takes part in driving Schwann cell proliferation *in vivo* (D’Antonio et al., 2006a). On the other hand, the rate of normal developmental Schwann cell death is also reduced. The result is that in developing nerves, abolishing TGFβ signaling does not materially affect Schwann cell numbers.

When TGFβ alone is applied to Schwann cells in culture, it induces apoptosis, but when this factor is applied in medium containing NRG1, TGFβ induces DNA synthesis (Parkinson et al., 2001; D’Antonio et al., 2006a). Therefore, it has been suggested that in developing nerves, the role of TGFβ is to amplify the proliferation of cells with tight axonal contact (and therefore exposure to axonal NRG1), while promoting the death of cells with a less effective axonal association (D’Antonio et al., 2006a).

#### The Hippo Pathway

Yap and Taz, key components of the Hippo signaling pathway, are expressed in Schwann cells. Genetic inactivation of either of these factors does not substantially affect early Schwann cell development. Combined inactivation of both Yap and Taz, however, results in a large reduction in proliferation of iSch, in addition to causing major changes in subsequent Schwann cell development including radial sorting and myelination. Yap and Taz exert their control over Schwann cell proliferation *via* the transcription factor TEA domain family member 1 (TEAD1; Poitelon et al., 2016; Deng et al., 2017; Grove et al., 2017).

#### The cAMP Pathway

In Schwann cells, genetic inactivation of the R1A regulatory subunit of protein kinase A, a key down-stream mediator in the cAMP signaling pathway, leads to severe drop in proliferation (Guo et al., 2013). The G protein-coupled receptor (GPR)126 is linked to cAMP elevation in Schwann cells and implicated in the control of Schwann cell proliferation (Monk et al., 2009, 2015). Also in cell culture, agents that mimic or moderately elevate cAMP levels drive Schwann cell division provided they are applied in combination with serum or defined growth factors such as NRG1 (Morgan et al., 1991; Monje et al., 2009; Arthur-Farraj et al., 2011). If cAMP levels are increased in such cultures, the mitogenic effect of cAMP gradually gives way to cAMP-induced activation of myelin genes (Arthur-Farraj et al., 2011). In a defined medium without growth factors, the effect of cAMP is primarily that of up-regulating myelin genes without significant stimulation of cell division (Morgan et al., 1991).

## Schwann Cells in Postnatal Nerves

The details of postnatal Schwann cell development and the biology of Schwann cells in older nerves have been the subject of numerous reviews, and lie outside the scope of this article (e.g., Jessen and Mirsky, 2005a, 2008, 2016, 2019; Taveggia et al., 2010; Glenn and Talbot, 2013; Jessen et al., 2015b; Monk et al., 2015; Salzer, 2015; Feltri et al., 2016; Boerboom et al., 2017; Ma and Svaren, 2018; Stassart et al., 2018; Jessen and Arthur-Farraj, 2019).

Briefly, all iSch in perinatal nerves are thought to have the same developmental potential. Some of them randomly come to assume a 1:1 relationship with larger diameter axons due to morphogenetic movements within the Schwann cell families (see “The Architectural Transformation of Nerve: the Generation of Connective Tissue” section; Figure 4B), referred to as radial sorting. Signals from the axons and the surrounding basal lamina activate the myelin program in these cells and drive them to assume the myelin phenotype, forming the electrically insulating myelin sheaths, required for fast conduction of action potentials. The smaller axons remaining in the families gradually assume a position in shallow troughs in the surface of Schwann cells thereby forming the unmyelinated Remak fibers.

The induction of Schwann cells to myelinate represents a striking example of cell extrinsic signals, in this case from axons and basal lamina, controlling phenotypic transformation during development. The fact that the highly specialized myelin Schwann cell phenotype generated through these interactions is not stable, but can be readily dismantled is another feature of particular interest in this system. This takes place in demyelinating neuropathies, and after nerve injury when axons degenerate, and myelin (and Remak) cells respond to the loss of axonal contact and other injury-related disturbance by transforming to an alternative Schwann cell phenotype that promotes nerve regeneration, repair Schwann cells (see “The Plasticity of the Schwann Cell Phenotype” section).

The remarkable plasticity of Schwann cells, their dependence on environmental signals and ability to adapt to the needs of the tissue they find themselves in, is underscored by the fact that when regeneration is accomplished and repair cells are no longer needed, they obey axon-associated signals to transform back to assume the myelin and Remak phenotypes necessary for normal nerve function.

## Conclusions

Glial cells are a central cellular component both of the PNS, where these cells develop form the neural crest, and the CNS, where they develop from the neuroepithelium. Notably, the key early stage in the development of both of these glial lineages, the SCP in the PNS and the radial glia in the CNS, share the stem-cell-like feature of developmental multipotency. At the same time, these cells show an obvious glial phenotype in terms of molecular, cell biological and structural features. Thus, SCP, like the iSch they give rise to, are embedded among, and ensheath axons, are strikingly dependent on axon-associated signals for development, and express a number of molecular markers associated with glial cells. At the same time, some of these cells contribute to the generation on fibroblasts, melanocytes and autonomic neurons during embryonic development, in addition to their major fate, the conversion to iSch. This transition takes place in late embryonic nerves, while postnatally iSch diverge to generate the myelin and Remak Schwann cells essential for the function of adult nerves.

These postnatal Schwann cells states retain a high degree of plasticity. For instance, during the normal physiological response to injury *in vivo* Schwann cells temporarily assume a repair Schwann cell phenotype that is crucial for regeneration, and as nerves regenerate former myelin cells can convert to Remak cells and vice versa. However, there is no clear evidence yet that under physiological circumstances Schwann cells can revert to the SCP stage, or that SCP—like cells take part in the injury response of peripheral nerves. Since SCP also have not been demonstrated in adult nerves, this cell appears to be unique to developing embryonic nerves.

In recent years, important transcription factors, epigenetic mechanisms and cell-extrinsic signals that control major events in the Schwann cell lineage have been identified. Nevertheless, significant gaps remain, particularly regarding our knowledge of the molecular controls of early events in embryonic nerves, such as the generation of SCP, lineage choice by SCP, and the architectural nerve transformation that takes place at the SCP/iSch transition.

## Author Contributions

KJ and RM jointly conceived the content and wrote the article.

## Conflict of Interest Statement

The authors declare that the research was conducted in the absence of any commercial or financial relationships that could be construed as a potential conflict of interest.
